# Integrating People, Context, and Technology in the Implementation of a Web-Based Intervention in Forensic Mental Health Care: Mixed-Methods Study

**DOI:** 10.2196/16906

**Published:** 2020-05-26

**Authors:** Hanneke Kip, Floor Sieverink, Lisette J E W C van Gemert-Pijnen, Yvonne H A Bouman, Saskia M Kelders

**Affiliations:** 1 Centre for eHealth and Wellbeing Research Department of Psychology, Health and Technology University of Twente Enschede Netherlands; 2 Department of Research Transfore Deventer Netherlands; 3 Faculty of Medical Sciences Universitair Medisch Centrum Groningen Groningen Netherlands; 4 Optentia Research Focus Area North-West University Vanderbijlpark South Africa

**Keywords:** eHealth, blended care, implementation, log data, forensic mental health care

## Abstract

**Background:**

While eMental health interventions can have many potential benefits for mental health care, implementation outcomes are often disappointing. In order to improve these outcomes, there is a need for a better understanding of complex, dynamic interactions between a broad range of implementation-related factors. These interactions and processes should be studied holistically, paying attention to factors related to context, technology, and people.

**Objective:**

The main objective of this mixed-method study was to holistically evaluate the implementation strategies and outcomes of an eMental health intervention in an organization for forensic mental health care.

**Methods:**

First, desk research was performed on 18 documents on the implementation process. Second, the intervention’s use by 721 patients and 172 therapists was analyzed via log data. Third, semistructured interviews were conducted with all 18 therapists of one outpatient clinic to identify broad factors that influence implementation outcomes. The interviews were analyzed via a combination of deductive analysis using the nonadoption, abandonment, scale-up, spread, and sustainability framework and inductive, open coding.

**Results:**

The timeline generated via desk research showed that implementation strategies focused on technical skills training of therapists. Log data analyses demonstrated that 1019 modules were started, and 18.65% (721/3865) of patients of the forensic hospital started at least one module. Of these patients, 18.0% (130/721) completed at least one module. Of the therapists using the module, 54.1% (93/172 sent at least one feedback message to a patient. The median number of feedback messages sent per therapist was 1, with a minimum of 0 and a maximum of 460. Interviews showed that therapists did not always introduce the intervention to patients and using the intervention was not part of their daily routine. Also, therapists indicated patients often did not have the required conscientiousness and literacy levels. Furthermore, they had mixed opinions about the design of the intervention. Important organization-related factors were the need for more support and better integration in organizational structures. Finally, therapists stated that despite its current low use, the intervention had the potential to improve the quality of treatment.

**Conclusions:**

Synthesis of different types of data showed that implementation outcomes were mostly disappointing. Implementation strategies focused on technical training of therapists, while little attention was paid to changes in the organization, design of the technology, and patient awareness. A more holistic approach toward implementation strategies—with more attention to the organization, patients, technology, and training therapists—might have resulted in better implementation outcomes. Overall, adaptivity appears to be an important concept in eHealth implementation: a technology should be easily adaptable to an individual patient, therapists should be trained to deal flexibly with an eMental health intervention in their treatment, and organizations should adapt their implementation strategies and structures to embed a new eHealth intervention.

## Introduction

Mental health issues cause an increasing number of personal, social, and financial burdens [1] and form a growing challenge for health care systems [2,3]. Technology can be used to address this challenge by supporting treatment of mental health problems in an efficient manner [3,4], while maintaining comparable clinical outcomes as standard in-person treatment [5-7]. The application of technology in mental health care is often referred to as eMental health: the use of technology for treating or preventing mental health disorders [8]. Multiple types of technology can be used. Multimodal web-based interventions based on cognitive behavioral therapies have been studied most often; other examples are mobile apps or virtual reality [8-11]. eMental health technologies can be used as a stand-alone tool, used individually by a person, but often they are integrated within in-person treatment, delivered by one or more therapists. The combination of offline, in-person treatment and online technologies in mental health care is referred to as blended care [12]. Blended care can offer various advantages. Among other things, it has the potential to increase patient engagement and sense of ownership for their treatment, reduce barriers toward receiving mental health care, offer treatment in a more standardized, evidence-based manner, and save time and decrease costs; it can also be personalized to optimally fit patients [4,8,13-15]. However, while eMental health has a broad range of potential benefits, most are not observed in practice [8,16].

An important reason for this gap between the potential and the current situation can be found in issues related to implementation. Implementation of eHealth (electronic health) refers to the strategies that are undertaken to realize the adoption, dissemination, and integration of eHealth innovation into care [17,18]. Examples of such implementation strategies are training and education of stakeholders, changing an organization’s infrastructure, using evaluative strategies, or supporting clinicians in using the intervention [19]. Ideally, these implementation strategies have a positive impact on implementation outcomes, defined in Table 1 [20,21]. However, studies show a broad range of issues with implementation outcomes for eMental health interventions, including acceptance by therapists and patients [22], therapists’ lack of knowledge on how to optimally combine eMental health and in-person treatment [14], a suboptimal fit with existing technologies such as electronic patient records, and practical barriers such as continuous maintenance of the technology or good internet access [16]. Consequently, to further actualize the benefits that eMental health can offer, implementation strategies should be improved. In order to identify relevant points of improvement, a recent review of eHealth implementation recommended that there is a need for more studies that critically analyze implementation strategies and outcomes of eMental health technologies in practice [23].

**Table 1:** Implementation outcomes and their definitions, adapted from Proctor et al [21].

Implementation outcome	Definition
Acceptability	Intervention is agreeable, palatable, or satisfactory among implementation stakeholders
Adoption	Intention, initial decision, or action to try or employ an intervention by a care provider or organization
Appropriateness	Perceived fit, relevance, or compatibility of the intervention for a given practice setting, provider, or consumer and/or perceived fit of the innovation to address a particular issue or problem
Cost	Cost impact of an implementation effort, dependent on the costs of the intervention, implementation strategy used, and location of service delivery
Feasibility	Extent to which a new intervention can be successfully used or carried out within a given setting
Fidelity	Degree to which an intervention was implemented as it was prescribed in the original protocol or as it was intended by the program developers
Penetration	Integration of an intervention within a service setting and its subsystems
Sustainability	Extent to which a newly implemented intervention is maintained or institutionalized within a service setting’s ongoing, stable operations

Several studies have focused on this issue and identified barriers and facilitators for the use of eMental health in practice [14,24-26]. However, as a recent review pointed out, most of the studies that analyze implementation of eMental health focus on one level (eg, factors related to patients) [16]. In order to get a good grasp of implementation of eMental health, attention needs to be paid to other levels as well (eg, organizational [16] or policy levels [13]). These recommendations on eMental health are in line with more general implementation models and literature: implementation should be seen as a multilevel and complex process [27] that requires a holistic approach [28,29]. Implementation models like the consolidated framework for implementation research (CFIR) [30] and the nonadoption, abandonment, scale-up, spread, and sustainability (NASSS) framework [31] account for the dynamic interaction between different factors and emphasize the interrelationship between characteristics and perspectives of users, organizations, and the intervention itself. Consequently, analyzing and integrating characteristics and perspectives of users; the context in which the eHealth intervention will be used; and the content, design, and use of the technology itself is expected to result in a complete, realistic picture of the implementation process and outcomes [20,28-32].

In order to apply such a holistic approach to eHealth implementation, a combination of different types of data that provide insight into the different aspects of eHealth implementation is necessary. Collecting multiple types of data does justice to the dynamic, complex interaction between factors that influence implementation, as opposed to analyzing these factors separately [33]. Also, from a holistic point of view, implementation should be studied from multiple angles and perspectives to gain in-depth insight into the technology, context, and people involved [18,28]. To illustrate: if only quantitative data from questionnaires are used to analyze implementation, an in-depth understanding of the reasons for the use of eMental health might be lacking [24]. However, when only using qualitative methods like interviews, information might not be as reliable or objective as is necessary for a thorough analysis of implementation [34]. Consequently, a mixed-methods approach where different types of quantitative and qualitative data are triangulated does justice to the complex integration of factors related to people, technology, and context. This is required for wielding a holistic approach toward the evaluation of eHealth implementation [35-38].

This study applied a mixed-methods approach to the holistic evaluation of the implementation process and outcomes of a blended eMental health intervention introduced in routine care by an organization for forensic mental health care. This setting provides an interesting context to study implementation in practice. First, the evaluation of implementation processes of eMental health technologies that have been implemented in routine care by an organization is expected to result in more ecologically valid results, as opposed to technologies that are being used because of research-initiated studies [20,35]. Second, our study focused on the implementation of an online eMental health platform with multiple modules that has been used for over 4 years in an organization that offers forensic mental health care to both in- and outpatients, which is expected to provide novel insights into long-term implementation processes in practice. Third, forensic mental health care is a branch of mental health care that focuses on treatment of a broad range of in- and outpatients who have committed or were on the verge of committing an aggressive or sexual offense, partly caused by one or more psychiatric disorders [39]. Because of the complex nature of this type of mental health care, and because not much is known about implementation in this type of setting [40], forensic mental health care offers an interesting setting to study implementation strategies and outcomes. Consequently, the goal of our study was to apply a mixed-methods approach to the holistic evaluation of the implementation strategies and outcomes of an eMental health intervention in an organization that offers forensic mental health care. The main research questions are as follows:

- Which implementation strategies were employed by the organization?
- What are the implementation outcomes in terms of adoption, fidelity, and penetration of the eMental health intervention?
- How do therapists perceive and explain implementation strategies and outcomes in terms of factors related to context, technology, and people?

## Methods

### Design

**Figure 1:**
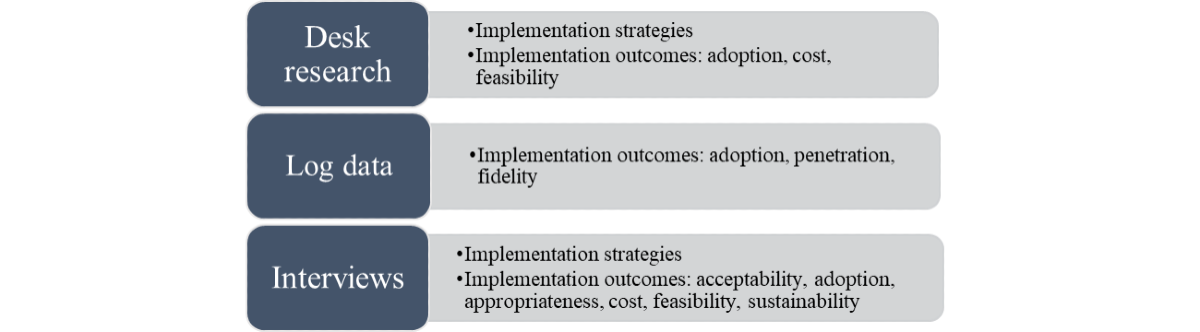
Overview of methods used in this study with types of outcomes seen in each method.

This mixed-methods study has evaluated the long-term use of a web-based application from multiple perspectives. A convergent parallel mixed-methods design was used [41] in which qualitative and quantitative data were collected in parallel, analyzed separately, and then merged. First, qualitative desk research was used to describe implementation strategies of the organization. Second, quantitative log data were used to analyze the objective use of an eMental health intervention by therapists and patients to gain insight into implementation outcomes. Third, interviews with therapists were conducted to gain more insight into implementation strategies and outcomes and analyze how they perceive and explain these strategies and outcomes. The purpose of this design is complementary [42]: the qualitative results are used to explain, illustrate, and provide more depth to the results from the quantitative log data, and the quantitative log data are used to enhance and illustrate the qualitative results in order to improve the interpretation of these findings and substantiate conclusions [43]. In order to answer the research questions, results were synthesized in the discussion by means of the aforementioned implementation strategies and outcomes. In Figure 1, an overview of this mixed-method study is provided.

### Setting

#### Organization

This study focused on the implementation strategies and outcomes of implementation of an eMental health intervention within one forensic mental health care organization. This organization started a pilot with the intervention in 2012 and gradually implemented the intervention in the entire organization around the beginning of 2014. The Dutch organization in which this study took place offers forensic mental health care to both in- and outpatients. From January 1, 2014, until May 30, 2019, 3865 in- and outpatients were treated at one of the locations of the forensic hospital. The hospital has two main outpatient clinics, where approximately 85% of patients are treated, and three main inpatient clinics, where the remaining 15% are treated. A total of 252 therapists worked at the hospital between 2014 and 2019.

Electronic patient records show that from January 1, 2014, until May 30, 2019, 2076 patients were treated in the outpatient clinic where the interview study took place, which is 54% of the total patient population of the forensic hospital. According to electronic patient records, 23.27% (483/2076) of the patients had a level of education of primary school or none at all, 22.74% (472/2076) attended secondary school, mostly vocational, 16.33% (339/2076) completed vocational secondary education, 3.32% (69/2076) completed higher secondary education at (applied) universities, and for 34.49% (716/2076), no information was available. Comorbidity was high in this patient population, and there was a broad range of diagnoses for psychiatric disorders (eg, personality, attention deficit, sexual, anxiety, depression, schizophrenia, and substance use disorders).

#### Online Intervention

The eMental health intervention that is the topic of this study is a website containing a collection of different types of modules. The intervention is suitable for all types of mental health care, not just forensic mental health care. The intervention was designed by a commercial company, and organizations that want to use it must pay for a subscription. In total, 234 modules were available in May 2019. These modules cover a broad range of topics including attention deficit hyperactivity disorder (ADHD), autism, social skills, mindfulness, personality disorders, trauma, addiction, and relaxation. The intervention also contains 6 modules specifically developed for forensic mental health care. These modules focus on creating offense chains and prevention plans, patient recovery, positive self-image, and leading a meaningful life. However, since forensic patients suffer from a broad range of psychiatric disorders and psychosocial problems, other, nonforensic modules are often suitable as well. Therapists must choose which module they find most fitting for their patient; they are able to assign any of the 234 modules. If a therapist does not assign a module, a patient is not able to use the intervention.

Each module consists of multiple sessions provided in a fixed order and accessed via a browser. These sessions consist of a combination of elements (eg, written information about the topic, a story from a peer (in video or text), written assignments derived from cognitive behavioral therapy, and videos to provide additional information about the topic of the session). The underlying assumption is that a patient must complete all sessions in order to be adherent to a module. In our study, the intervention is used as part of blended care, which means that the patient is asked to complete assignments in each session on which the therapist provides written feedback. The patient can only continue with the module once the therapist has provided feedback on a session.

### Desk Research

In order to identify the implementation strategies employed by the organization, desk research was conducted. In total, 18 documents describing the pilot project and implementation of the eMental health intervention were obtained from a policy advisor of the forensic organization who has been involved in the implementation of the intervention from the start. Examples of included documents are reports on the planning, progress, and outcomes of the pilot; communication with management; and brief research reports. In order to summarize the implementation process, a timeline with a chronological description of decisions, products, and events was distilled.

### Log Data Analysis

Log data from the entire organization from December 2013 until May 2019 were collected and analyzed. These data were analyzed to gain insight into the following implementation outcomes: adoption by therapists and patients, fidelity, and penetration of the eMental health intervention in the organization. Log data refers to anonymous records containing information of every action performed by every user [36]. To be able to analyze the log data, several files with anonymized log data were retrieved from the platform. First, multiple files with information on modules assigned to patients and sessions completed were downloaded. The raw data were combined and organized into an overview of modules and accompanying lessons by means of a macro in Excel (Microsoft Inc). Second, a file with the monthly number of feedback messages sent by individual therapists was retrieved. All log data were stored and processed anonymously and in line with privacy regulations relevant at that point in time. Ethical approval (No. 18408) was obtained from the ethics committee of the Faculty of Behavioral, Management, and Social Sciences from the University of Twente.

### Interview Study

#### Participants

In order to gain a deeper insight into how therapists perceive and explain implementation strategies and outcomes, interviews were conducted with therapists working at one outpatient clinic of the forensic hospital. In this clinic, therapists were expected to use the eMental health intervention. Therapists were interviewed because of their key role in implementation: if they did not introduce the intervention to the patients, patients could not participate. The attitudes and actions of health care professionals appear to have an essential role in eHealth implementation [44]. At the time of the interviews, 20 therapists were working at the outpatient clinic. All therapists were invited to participate by the manager of the outpatient clinic, but two of them were excluded because they did not receive training and had no experience with the eMental health intervention.

#### Materials and Procedure

The main goal of the interview study was to identify factors which, according to therapists, are related to the use and nonuse of the eMental health intervention. These factors provide insight into implementation strategies and outcomes. In order to achieve this, semistructured interviews with the 18 therapists were conducted in April and May 2018 by two researchers (KR & NtC) at the outpatient clinic. The interviews were audiorecorded and took between 21 and 61 minutes, with an average of 41 (SD 10) minutes. Ethical approval (No. 18239) for the interview study was given by the ethics committee of the Faculty of Behavioral, Management, and Social Sciences of the University of Twente.

The interview started with a brief explanation of the goal and content of the study. After that, informed consent was signed. The interview scheme consisted of 6 main categories with accompanying open questions. First, sociodemographic questions were asked. Second, experiences with the introduction of the eMental health intervention were discussed. Third, the participant was asked to describe in what way, how often, and with which patients he or she used the eMental health intervention. Reasons for nonadherence were also discussed. The fourth part contained questions on the potential and experienced added value of the eMental health intervention for the therapist, patient, and organization. Fifth, participant was asked to describe what the ideal situation with regard to the use of the eMental health intervention would look like. In the sixth part, barriers for using the intervention were discussed. These questions were divided into 5 topics, loosely based on 5 relevant domains of the NASSS framework [31]: barriers related to patients, therapists, and the forensic health care organization; the wider context; and characteristics of the eMental health intervention. The NASSS framework was used because its holistic nature, in which attention is paid to different types of factors and their interrelationships, fits the research goal of this study. The interview’s final question focused on what should be done to overcome these barriers and optimize benefits.

#### Analysis

The interviews were transcribed verbatim. In order to answer the research questions, deductive, top-down coding via the NASSS framework was combined with an inductive, bottom-up analysis of all fragments belonging to a domain of the NASSS framework. First, all relevant fragments were analyzed deductively by categorizing them into 1 of the 7 domains of the NASSS framework. This deductive analysis ensured a clear main structure of the results in line with the holistic focus of the research goals. The NASSS framework was used to structure the analysis because of its focus on technology in health care and holistic approach [31]. After deductive analysis using domains of the NASSS framework, fragments within each domain were analyzed inductively to look for more specific factors important for the use of the technology according to the interviewed therapists. A coding scheme was iteratively created based on all fragments of the first 5 interviews by one researcher (HK) via the method of constant comparison [45]. Using this coding scheme, the 165 fragments of these first 5 interviews were independently analyzed by a second researcher (FS) to determine interrater reliability of the coding scheme. The joint probability of agreement was 89%. After deliberation of the fragments that were assessed differently by the researchers, agreement was reached on all fragments, and several definitions of the code scheme were fine-tuned. No further adaptations to the underlying structure of the code scheme were required. Because of the high interrater reliability, one researcher (HK) coded the remaining 572 fragments and discussed them with the other researcher (FS) in case of doubt. Again, definitions of codes were adapted throughout the process.

### Synthesis

In this mixed-methods study, results were synthesized via the implementation strategies and outcomes. Figure 1 shows which method was used to provide information for which implementation outcome. To synthesize the results, implementation strategies were summarized using all three methods. Also, the most important findings per implementation outcome were described and supported by outcomes of desk research, log data analyses, and codes that arose from the inductive analysis of the interviews.

## Results

### Desk Research

**Figure 2:**
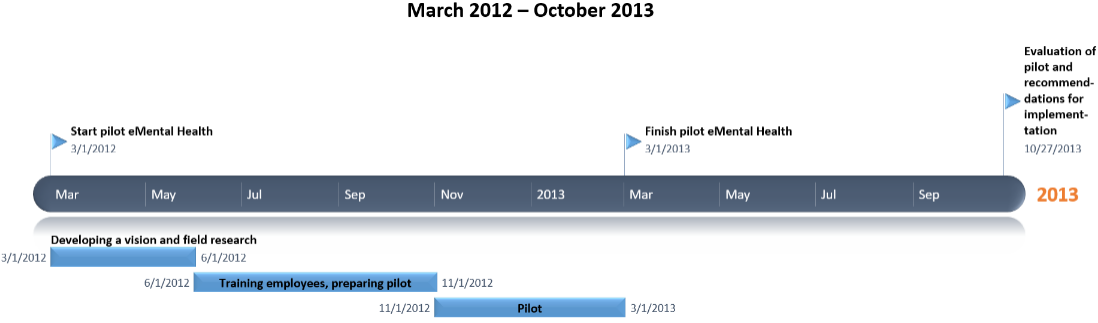
Timeline of the pilot phase of the online intervention.

In order to describe the implementation strategies, desk research was conducted with documents generated by the organization. Before the online intervention was disseminated throughout the organization, a pilot was conducted in which the intervention was used on a small scale. This pilot was coordinated by a project team consisting of therapists and policy advisors, and its timeline is visualized in Figure 2. The goal of the pilot was to improve the content and usability of the intervention and develop a good strategy regarding communication about the eMental health intervention to the organization. The pilot started with an exploratory phase, after which 90 employees were trained and instructed to use the intervention for several months. Desk research did not show how many therapists and patients participated in the pilot. The experiences of the pilot were used to create a strategy and recommendation for implementation of the intervention in the organization. Example of recommendations that arose from the pilot were to provide all employees with the training that participants of the pilot received and write guidelines on how to embed the intervention’s modules in existing care programs.

**Figure 3:**
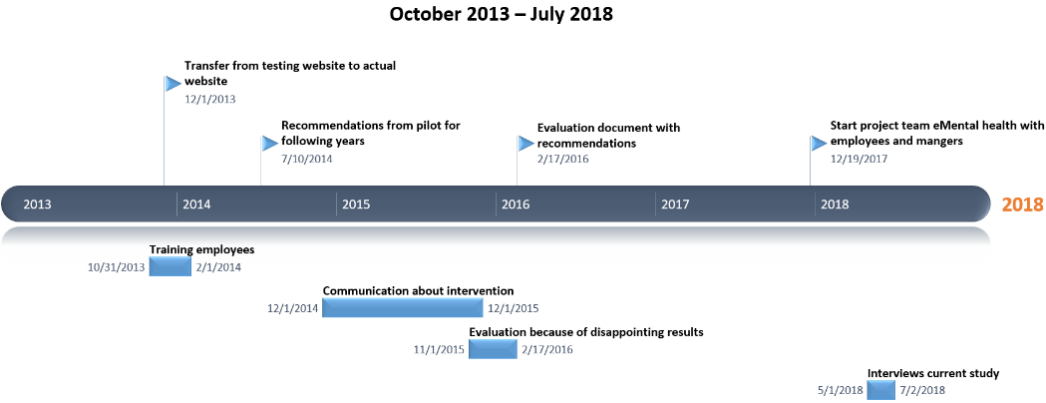
Timeline of implementation strategies for the online intervention.

After the pilot, the online intervention was introduced to all therapists of the organization. An overview of the timeline, until July 2018 when the interview study of this paper was finalized, is provided in Figure 3. The main goal of the implementation was to fully integrate the eMental Health intervention in all primary and supportive processes. Consequently, all therapists received training and were expected to use the intervention. However, because use in practice was not as high as expected, an evaluation was conducted in 2016 that resulted in several recommendations, including that management should improve communication about targets of the eMental health intervention to therapists, a clear overview of useful modules should be created, some skilled therapists should be appointed as champions who can support colleagues in using the intervention, and therapists should motivate patients more by, for example, calling them if they did not use the intervention. When the recommendations did not lead to any major improvements, a new project team to installed to improve implementation in 2017; that team initiated this study.

### Log Data Analysis

#### Patients

In order to gain insight into implementation outcomes, log data that provide insight into patient use of the intervention were analyzed. From December 2013 until May 2019, 721 unique patients were assigned to at least one module of the eMental health intervention by their therapist. In total, 1019 modules were assigned to these 721 patients. Most patients (514/721, 71.3%) were assigned 1 module, 16.8% (121/721) were assigned 2 modules, 6.4% (46/721) were assigned 3 modules, and 2.5% (18/721) worked on 4 modules. The remaining 2.8% (20/721) worked on 5 to 10 modules. Finally, there were 2 patients who worked on many different modules: one patient worked on 23 modules and another patient on 28. Of the patients, 18.0% (130/721) fully completed at least 1 module, 50.6% (365/721) completed 1 or more lessons but did not complete at least 1 module, and 30.0% (216/721) patients did not complete any lessons at all.

In total, 98 different modules were assigned to patients. The median number of patients assigned to an individual module was 4. The offense script and prevention plan module was assigned to the most patients (104/721), and 18 modules were assigned to only 1 patient. Table 2 provides an overview of all modules that were assigned to at least 10 patients, including an overview of how many patients completed all lessons of the module, completed 1 or more lessons, or did not complete any lessons. When looking at all modules and patients, 180 of the 1019 modules (17.66%) were completed, meaning that all lessons were finished. For 448 of the 1019 modules (43.96%) at least 1 lesson was finished, but not the entire module. On average, when a module was started but not completed, 43.17% (2155/4992) of the modules’ lessons were completed. When looking at the longer modules containing 10 to 26 lessons, 44.43% (1994/4488) of the lessons were completed. Of the shorter modules with 9 or fewer lessons, 40.28% (203/504) of the lessons were completed. Finally, in 412 of the 1019 modules (40.43%), no lesson was finished.

**Table 2:** Overview of the total and relative number of patients that completed, didn’t complete, or partially completed modules that were assigned to at least 10 patients.

Topic of module	# lessons	Module completed, n (%)	Module not completed, ≥1 lesson finished, n (%)	Module not completed, no lessons finished, n (%)
Offense script and prevention plan (n=104)	25	14 (13)	71 (68)	19 (18)
Aggression (n=94)	14	7 (7)	48 (51)	39 (41)
Autism psychoeducation (n=76)	10	12 (16)	36 (47)	28 (37)
Substance abuse problems (n=63)	15	5 (8)	37 (59)	21 (33)
Mindfulness (n=59)	9	6 (10)	32 (54)	21 (36)
Offense script and prevention plan (short version; n=55)	17	12 (22)	34 (62)	9 (16)
Expert of yourself (n=53)	10	11 (21)	26 (48)	16 (30)
ADHD^a^ (adults): understand your ADHD (n=40)	3	11 (28)	9 (23)	20 (50)
Thought scheme (n=29)	2	9 (31)	10 (34)	10 (34)
Skills for mild intellectual disorders (n=22)	9	3 (14)	11 (50)	8 (36)
Loved ones of patients (n=20)	9	3 (15)	11 (55)	6 (30)
Forensic: positive self-image (n=18)	4	9 (50)	3 (17)	6 (33)
Social skills (n=17)	1	5 (29)	5 (29)	7 (41)
Social skills: saying no (n=15)	2	6 (40)	5 (33)	4 (27)
Information on psychotic disorders (n=14)	9	5 (36)	7 (50)	2 (14)
Psychoeducation for personality disorders (n=14)	4	3 (21)	2 (14)	9 (64)
Generalized anxiety (n=12)	9	2 (17)	7 (58)	3 (25)
ADHD (adults): I want to think before I act (n=11)	9	6 (55)	0 (0)	5 (45)
ADHD (adults): I want to clear my mind more (n=10)	1	2 (20)	0 (0)	8 (80)
Aggression in your relationship (n=10)	14	0 (0)	7 (70)	3 (30)

^a^ADHD: attention deficit hyperactivity disorder.

#### Therapists

Therapists’ use of the intervention was analyzed as well to gain insight into implementation outcomes. A main task of the therapist in using the eMental health intervention was to give feedback on patient assignments. A patient could only continue with the next lesson once the therapist provided feedback, and all lessons required feedback. In total, 172 therapists had accounts, which means they could use the intervention and provide feedback. The median number of feedback messages sent per therapist was 1, with a minimum of 0 and maximum of 460. Of the 54.1% (93/172) of therapists who gave feedback from January 2014 to May 2019, 25.0% (43/172) gave feedback 1 to 5 times, 25.0% (43/172) gave feedback 6 to 19 times, 25.0% (43/172) gave feedback 20 to 50 times, and 25.0% (43/172) gave feedback 51 to 460 times. Table 3 shows how many therapists sent how many feedback messages, showing one major outlier who gave feedback 460 times. The therapist who gave the second highest amount of feedback sent 251 messages.

**Table 3:** Number of feedback messages sent by therapists.

Messages sent	Number of therapists
0-35	144
35-70	12
70-105	7
105-140	2
140-175	0
175-210	4
210-245	1
245-280	1
280-315	0
315-350	0
350-385	0
385-420	0
420-455	0
455-490	1

**Figure 4:**
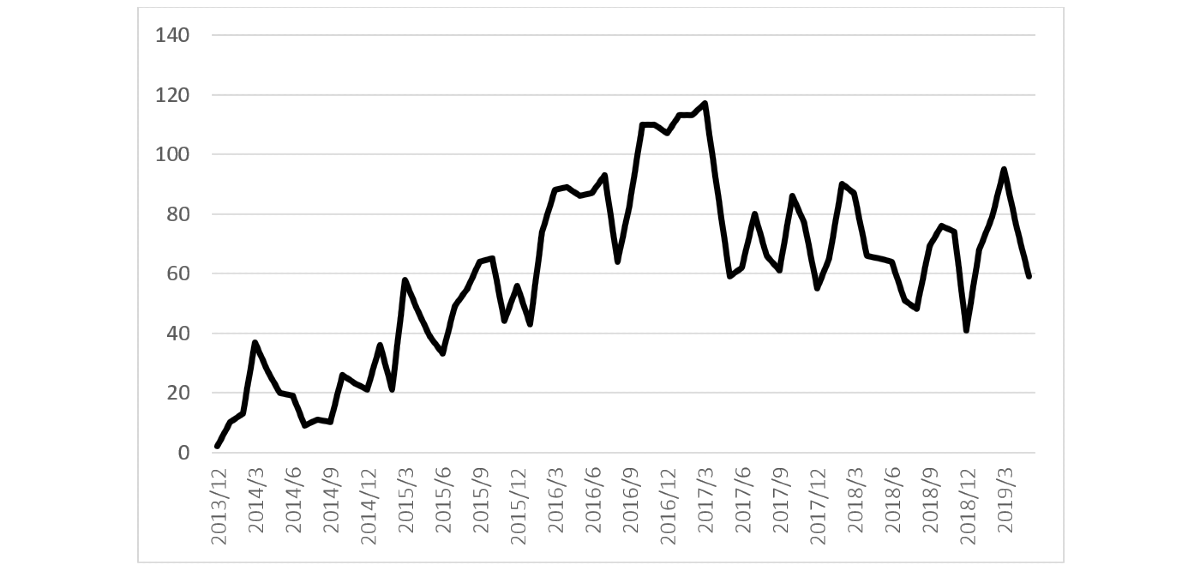
Number of feedback messages sent by therapists over time.

Finally, Figure 4 shows the total number of feedback messages that were sent over time, from the introduction of the eMental health intervention until May 2019. The figure shows an increase in sent feedback messages with a peak in 2016, after which the number of messages decreased and seemed to stabilize at approximately 60 messages per month.

### Interviews

#### Participants

In order to gain insight into factors related to implementation outcomes, 18 therapists from one outpatient clinic were interviewed. The therapists had different occupations: 8 psychologists, 6 social workers, 2 system therapists, 1 trauma therapist, and 1 forensic nurse were interviewed. Participants had an average age of 42.5 (SD 10.46) years with a range of 28 to 60 years, and 10 were female. At the time of interviewing, they had been working in forensic care for an average 13.18 (SD 8.68) years with a range from 8 months to 29 years.

#### First Impressions, Introduction, and Subjective Use of the Intervention

When asked about their first impression of the eMental health intervention and its introduction by the organization, most therapists were positive, as can be seen in Table 4.

**Table 4:** Therapist responses to survey questions.

Survey question	Very bad	Bad	Neutral	Good	Very good
First impression of the intervention	0	0	6	11	1
Introduction to the organization	0	1	5	10	2

When asked about use of the intervention, 4 therapists indicated that they received training but never used the intervention with a patient. The other 14 therapists had used the intervention with on average 8 patients, with a minimum of 2 and a maximum of 15. Therapists used the intervention in different ways in their in-person sessions with patients. Some discussed the assignments completed by patients very irregularly or never, whereas others discussed them structurally in each session. All therapists saw the intervention as an addition to treatment and used it in a blended manner, where in-person contact was seen as more important. Almost all therapists indicated that most patients did not finish modules and it was hard to keep them motivated.

#### Coding Schemes

##### Overview of Codes

The NASSS framework was used to structure the interview scheme and guide the coding process. Consequently, the main codes that were identified in the interviews are mostly aligned with the domains of the NASSS framework, as can be seen in Table 5. However, since a combination of deductive and inductive coding was used, there are several differences between the codes and domains of the NASSS framework. First of all, the adopter domain was split into a therapist and patient code. Integrating all these subcodes into one main adopter code would not have done justice to the differences between these types of factors. Furthermore, the embedding and adaptation over time domain of the NASSS model was not identified in interviews because therapists had difficulties providing a long-term vision on the technology and organization.

**Table 5:** Main codes, their definitions, number of interviews quote was mentioned (Nint), and number of times code was mentioned (Ntot).

Main code	Definition	N_int_	N_tot_
Adopters–therapists	Characteristics, cognitions, or behaviors of therapists that influence the use of technology in treatment	18	203
Adopters–patients	Characteristics, cognitions, or behaviors of patients that, according to therapists, influence the use of technology in treatment	18	194
Value proposition	The (desired) added value that a technology has or should have for treatment, according to therapists	18	121
Technology	Influence of the technology’s features on use by therapists and patients	18	104
Organization	Characteristics, culture, and activities of the organization that influence use of technology by therapists	18	94
Wider system	Influence of activities by the broader context on use of technology by organizations and therapists	11	7
Condition	Extent to which the nature of the patient’s condition (or disorder) influences use of technology, according to therapists	3	4

##### Adopters–Therapists

This main code was mentioned most often and refers to characteristics, cognitions, or behaviors of therapists that influence the use of eHealth in treatment. This code was found in all interviews, and 203 fragments belonging to this main code were identified. The subcodes are reported, defined, and illustrated with one or two quotes in Table 6. As can be seen in the table, therapists discussed a broad range of factors related to themselves that could influence the intervention’s use. The most mentioned code referred to them not putting in enough time or effort to start or keep using the intervention. This could result in a lack of knowledge and skills to optimally use the intervention. The perceived lack of time was partly attributed to high workload but also to lack of enthusiasm to use the intervention. Among other things, not all participants were keen to work with technology in general. Several therapists believed that the intervention did not have enough benefits for their patients, and some felt that the technology was not easy to embed within standard treatment. Related to this, many therapists indicated that using the intervention was not in their system: while they often were willing to try it, most of the time they simply forgot about it because it was not part of their treatment routine. Consequently, many therapists did not introduce the intervention to their patients or did not motivate them enough to keep using it. Because the intervention was often not on the top of their minds, therapists indicated that they hardly discussed it with their colleagues. Finally, according to several participants, an important reason for successful use was having experienced benefits of the intervention for the patient: if the intervention fits an individual patient’s skills and problems, chances on successful use were said to be higher.

**Table 6:** Subcodes of the main code “adopters–therapists,” their definitions, illustrative quote, number of interviews the quote was mentioned (N_int_), and number of times code was mentioned (N_tot_).

Subcode	Definition	Illustrative quote	N_int_	N_tot_
Investing effort and time	Effort and time the therapist is able and/or willing to invest in getting acquainted with and structurally using the technology	*You have to know what happens in the module, what it contains. And then you have to check which assignments have been completed, and then give feedback on it. That costs extra time. Perhaps that’s why people are hesitant. (pp. 13)* *It can’t be like you saying: “Hey, just fill it in, but I’ll never look at it again.” You have to make time to thoroughly look into it and to familiarize yourself with it. (pp. 4)*	18	48
Supporting patients	Extent to which a therapist actively introduces the technology to patients and/or tries to motivate and support patients to keep using it	*Sometimes I know in advance: “This one will not use it,” and maybe that’s a self-fulfilling prophecy, that might be. But still, I’m not really inclined to start then. (pp. 7)* *Because I’ve tried a lot of times to start with a certain module with patients who are not passionate about it. [...] But I notice it with myself: because the passion is there I can often make it work. So that’s very necessary. (pp. 12)*	17	37
Integration of technology in routines	Extent to which the technology is integrated in the therapist’s routine and/or whether they automatically think of using the technology	*Yeah, I don’t know what’s the reason, I cannot really explain it, but despite that I like the modules, it’s not on the top of my mind. So there is something that’s stopping me from diving into my computer to find out which module fits with a patient. And I don’t really know why it doesn’t come up. (pp. 15)* *Well, I agreed with a patient that they would hand it in next week Tuesday. Well ok, but then I didn’t receive it and I forgot about it myself. The patient stopped doing it and well, it kind of disappears into the background. I now resolved it by planning appointments in my calendar, so that I’ll be reminded to send reminders to patients. (pp. 6)*	16	36
Knowledge and skills	Therapist’s level of knowledge about the technology and skills to appropriately use it in treatment	*I also find it difficult to figure out which module fits which patient. [...] And I think that in my case, I’d just have to know which modules exist. And then I can just say “This fits you nicely, I’d like to recommend this, see if it’s possible” and then get to work. (pp. 7)*	13	34
Attitude toward the technology	Therapist’s opinion on and feelings toward using technology in treatment	*I think it depends on in which generation you’ve grown up, I think younger colleagues have more feeling with it. [...] and that asks for a considerable adjustment, transformation, also for a number of older colleagues. (pp. 1)* *And working with people, I still like doing that. Except for all that technical fuss, computers mean nothing to me, and also smartphones... I don’t have any feeling with that and experience it as a burden. (pp. 2)*	12	28
Discussing technology with colleagues	Technology as a topic of conversation among therapists inside and outside of official meetings	*Then I’ll say in our team: “I’m hearing this and this and this,” and sometimes someone in the team says: “Hey, eMental Health is something.” I think that if people in our team say “Maybe eMental health is an option” more often... And if that rhythm isn’t there, that’s the reason why there is very little eMental health. Or at least within our team. (pp. 12)*	10	21
Experienced benefits	Extent to which a therapist perceives that the technology has positive effects on the patient’s treatment outcomes	*So then we try to shape the treatment in another way, instead of continuing with something [the module] of which you have the idea that it doesn’t have that much effect. (pp. 3)*	2	4

##### Adopters–Patients

The main code “adopters–patients” was mentioned by all 18 therapists and refers to characteristics, cognitions, or behaviors of patients that influence the use of eHealth in their treatment. In total, 8 subcodes, presented in Table 7, and 194 fragments related to this main code were found. Therapists discussed multiple types of patient-related factors that, according to them, could influence the use of the intervention. It was frequently mentioned that many forensic psychiatric patients are often not motivated to start or keep working on the intervention. Therapists indicated that this was in line with low motivation for their treatment in general, which is partly due to the often obligatory nature of these patients’ treatment. Furthermore, therapists stated that a large share of the patient population has cognitive impairments due to psychiatric disorders—such as problems with focusing—or received very little education (eg, having finished only primary school). According to the therapists, this can cause problems with patients understanding the mostly text-based intervention, completing written assignments, being able to individually reflect on their behavior, and having the required technological skills to be able to practically use the intervention. Furthermore, patients have to work in the intervention individually in their own time, which some therapists compared with homework. Therapists stated that many patients have difficulty with this: a large share of the patient population was not seen as conscientious enough to independently work on the intervention. For example, patients often do not stick to agreements about when assignments were to be completed. Furthermore, therapists indicated that forensic psychiatric patients often have multiple psychiatric disorders and problems within their social environment, which might negatively influence their use of the intervention. When severe psychosocial issues occur, patients might be too preoccupied with these issues to use the intervention. Furthermore, not all patients have access to a computer or laptop, or they do not have a quiet place where they can comfortably work on the intervention.

**Table 7:** Subcodes of the main code “adopters—patients,” their definitions, illustrative quote, number of interviews quote was mentioned (N_int_), and number of times code was mentioned (N_tot_).

Subcode	Definition	Illustrative quote	N_int_	N_tot_
Motivation	Extent to which patient is motivated, enthusiastic, or open toward working with the technology in their treatment	*I kind of think that, I think I’ve mentioned before that if that motivation is present, if they have the feeling that eMental Health fits their problems. And that has to be the case. They don’t have to think: “Well, what about the problems I have....” They do have to see the connection to be motivated. (pp. 4)* *Because very demotivated patients who don’t want to be here anyhow... See, it’s easier to not work on eMental Health at home, then not attending a face-to-face appointment. They’d earlier drop the eMental health then not coming here. (pp. 9)*	14	40
Conscientiousness	Extent to which patient is diligent in working on the technology and fulfills commitments regarding the use of the technology outside of treatment	*Reading comprehension, making assignments, those kinds of things. If you’re not used to doing homework, if you’ve never done homework in your life—eMental Health is actually homework. So that’s a skill in itself. They have to be able to do it. (pp. 9)* *Next, we open the module and then it seems to go well for about 1 or 2 sessions. But then there’s always an “appointment forgotten” or “not finished” or those kind of things. After 3 months someone has stopped filling in the assignments, and only completed two sessions. (pp. 11)*	14	27
Literacy and educational level	Patient’s ability to write, read, and understand treatment-related information in the technology	*But I noticed that it wasn’t really working. We’ve done it a couple of times, but there were a lot of difficult words. And if I explain the words, he’ll forget what it means after that. (pp. 4)*	14	22
Experienced benefits	The extent to which a patient experiences a positive influence on his or her treatment because of the use of the technology	*And whether the module fits the treatment and problems of the patient. [...] If you, for example, have a module on autism, and someone recognizes himself in that, in a module on psychoeducation for example. Then it can actually be useful in their daily lives. (pp. 6)* *And also what I said earlier, that a module fits the needs of a patient. So if you use a sleeping module on someone with sleeping problems, chances are higher that he will continue with it. (pp. 16)*	14	22
Psychosocial situation	Level of stability of patient’s personal life and/or mental state that is required to use a technology	*I don’t really think about it with people who are in a crisis situation. Because in my experience, they really don’t have the need to discuss [the intervention]. You have to be in clearer waters, before they at least... Yes, that’s my experience, before you can make a connection with someone who’s overwhelmed by stress. If you, for example, have trouble about your social security benefit, or trouble with the neighbors, or trouble with your spouse, then it doesn’t really work for those people anymore. (pp. 10)* *Patients who are very prone to psychiatric crisis [...] or have so many psychosocial problems, well, then it won’t work. Then you’re only trying to stabilize them and you cannot use eMental health. (pp. 9)*	13	28
Technological skills	Level of practical skills required for successfully using information and communication technologies such as computers or smartphones	*And I also notice that with eMental Health, the problem is that if a patient doesn’t know anything about the computer, who isn’t focused on that, you won’t be able to make it work. You can jump high or low, but you won’t get it done. (pp. 12)*	12	17
Availability of technological resources	Patient’s access to necessary preconditions to use the intervention: technological device, appropriate working area, and good internet connection	*I think that in their own environment, where they like doing it. They have to be able to do it privately, not that there’s someone around the entire time. So privacy is important for them, I think. We can’t facilitate that; they have to arrange that themselves. Or we’d have to offer them a place to work here, so they can sit behind a computer here. (pp. 7)* *But I have had several people who were pretty positive about it, but who didn’t have a computer, or their computer broke. (pp. 8)*	11	18
Reflective skills	Patient’s ability to independently write about and reflect on emotions, cognitions, and behaviors in the technology	*I’m dealing with a lot of patients that find it really difficult to put their emotions and feelings on paper. They don’t talk about those things regularly, like conflicts with their wife. Those people just didn’t learn that. And yeah, if that’s already difficult in a conversation to tell what you feel, what you want, or what you want differently, it’s even more difficult to type it if you’re alone, sitting behind a computer. That’s on another level. (pp. 10)* *Because the simplest question—he understands them, but he said to me: “I simply cannot put it into words.” And if you have to do such assignments, and answer things... The fact that he hardly gives an answer, that partly has to do with him not being able to visualize and verbalize. (pp. 1)*	11	21

##### Value Proposition

The main code “value proposition” refers to the added value that a technology has or should have for treatment, according to the therapists. It was mentioned by all 18 therapists, 5 subcodes were identified, and 121 fragments were found in all interviews. As can be seen in the previous tables, not all therapists were positive about the intervention and did not use it often, but they were able to identify a broad range of potential and actual advantages of the eMental health intervention. These focused, among other things, on the content of the treatment: the intervention was said to have the potential to improve the quality of treatment by, for example, providing more structure to the treatment. Also, because therapists often also read the text of the intervention, several participants indicated that using the intervention might further improve or deepen existing knowledge about disorders. The intervention can also support patients in gaining new knowledge and skills (eg, new insights about a psychiatric disorder or an improvement of reflective or coping skills). Furthermore, several therapists explained that because patients must work on the intervention individually, their feeling of responsibility for their own treatment might increase, and they might ascribe positive changes more to themselves instead of their therapists. Moreover, several practical advantages were mentioned, among which saving time of therapists and patients because of less traveling time and replacing part of in-person treatment with the intervention, an increase of patients’ access to care because they can individually work on their treatment at their own pace, and providing a new way of delivering treatment to patients. The subcodes are presented in Table 8.

**Table 8:** Subcodes of the main code “value proposition,” their definitions, illustrative quote, number of interviews quote was mentioned (N_int_), and number of times code was mentioned (N_tot_).

Subcode	Definition	Illustrative quote	N_int_	N_tot_
Improving treatment	Possibility of technology to improve quality of and further structure of face-to-face treatment	*It does help you to focus the treatment on what someone needs. And it helps to not get bogged down in other, less relevant matters that people bring up, where it actually shouldn’t be about. Someone has a treatment goal and the program fits that goal and that is what you will be doing. So it really helps to frame your therapy. (pp. 11)* *Often, there are also things in there that I don’t have on the top of my mind. And sometimes it’s pretty nice to work via a protocol, that you encounter things of which you think: “Hey, I didn’t think about that at all!” (pp. 2)*	14	39
Practical benefits	Benefits for patients, therapists, and the organization related to practical matters such as time and money	*Well, I think it’s very nice that you can put patients to work at a time which suits you, and that they can work whenever it suits them. So planning appointments is less of a hassle. And it’s just like communication via WhatsApp or email: you all do it in your own time, so in that sense it’s easier insertable in everyone’s schedule. (pp. 16)* *Well, the patient can work on it at home. He’d have to come here less often, face-to-face. So you’d have to plan an appointment less often, which can enable you to see more other patients. (pp. 5)*	14	26
Increase of knowledge and skills	Possibility for the patient or therapist to acquire new insights and skills into the patient or the disorder	*An advantage for a patient with whom I have done the aggression module is that he did really gain more self-insight and came a little closer to himself. With the other patients I didn’t really have a hallelujah experience, but with him it seemed like the penny has dropped. (pp. 15)* *What I like about that is that you also go through the content yourself. [...] And I really like that of the modules, that you learn things from it yourself, and him as well. (pp. 12)*	13	22
Increase in patient independence	Enabling patients to work more independently and feel more ownership for their treatment	*Well, the fact that someone does it himself with the module, that they are prouder at themselves because they’ve achieved something. That it wasn’t the therapist who helped you, but that you’ve done it yourself. (pp. 16)* *If they’re in my room, I often have a fairly high pace, I am pulling and pushing them. But with the intervention, they’re in a calm environment and can think calmly. (pp. 2)*	13	19
More options for treatment	Possibility of technology to offer a broader range and different types of treatment to patients	*Well, for example, if a patient is working on the offense chain, you might notice that he has the need to practice more skills. Or wants to read a bit more about certain relaxation exercises. And if we’re not there yet in the module, I move these topics forward, so that we can work on those at that point in time. Or I check in another module if there’s something there, that they can work on a topic in between. [...] Then I think: “This fits well at this point in time,” and then we can continue with the treatment. (pp. 9)*	11	17

##### Technology

This main code was mentioned in all 18 interviews and focuses on the influences of the technology’s features on use by therapists and patients. In total, 3 subcodes and 104 fragments were identified, as can be seen in Table 9. Usability of the technology was often mentioned by therapists. While a few were fairly positive, most found the technology not easy to use for themselves or for patients: it did not fit their preferences and way of working. Furthermore, while most therapists were relatively positive about the intervention’s look and feel, it was mentioned that the way the content was presented was not very suitable for many patients, for example, due to a lot of text or too many sessions within modules.

**Table 9:** Subcodes of the main code “technology,” their definitions, illustrative quote, number of interviews quote was mentioned (N_int_), and number of times code was mentioned (N_tot_).

Subcode	Definition	Illustrative quote	N_int_	N_tot_
Ease of use	Extent to which therapists find use of the technology intuitive, clear, and structured	*I feel that the eMental health intervention is too big, or too fuzzy. As I just mentioned, all these modules, I know a few, but I think that there are a lot, also specified on other diagnoses. But I find it tricky to find these things. So I don’t think it’s very well arranged. (pp. 9)* *I’d see that as tiles in [the electronic patient record] which we are using currently. In User you have multiple tiles and the patient record, and it should also have a tile of [the intervention], on which you click and then you can start. [...] If there would be a block of [the intervention], it would be really easy to go to it. I think that would be more user friendly than when you have to go to the website yourself to log in, because then you have taken multiple additional steps. (pp. 7)*	16	68
Presentation of content	Therapist’s opinion on the ways in which the treatment-related content of the technology is presented to the patients	*The module itself should be shorter. Both the individual sessions and the number of sessions in a specific module I’d make shorter. I feel that certain explanations are too difficult for some patients. So I think there is not enough supply for people with a low intelligence, and a large number of our people has a lower than average intelligence. (pp. 18)*	8	21
Appearance	Therapist’s opinion on the overall look and feel of the design of the technology	*Because I think those modules are really cool. I’m thinking “Wow, the person that came up with this has it right!” But it’s just fresh, I’d almost say happy, but also friendly, and user-friendly. And if you can have these things together in a module text, with some videos and some other things and some explanation, that’s just amazing! (pp. 12)* *With regard to the design, with videos and images, it’s stimulating and appealing. It’s not a boring booklet that you hand out. (pp. 4)*	7	10

##### Organization

This main code refers to the characteristics, culture, and activities of the organization that influence the use of technology by therapists, and was mentioned in all 18 interviews. In total, 94 fragments for 4 subcodes were identified, which are explained in Table 10. Almost all interviewed therapists explained that the intervention was introduced to them by means of a course in which they gained practical skills to use it. According to therapists, the organization did not pay a lot of attention to the intervention after this course. Multiple therapists indicated that the intervention was often not discussed in official meetings. This was viewed as a partial explanation for therapists not remembering to use the intervention on a regular basis. Also, several therapists indicated that they did not experience enough support for questions about the content of the intervention or the way they could embed it in treatment. To illustrate, some therapists required more support when working with unmotivated patients or had questions about how to integrate assignments of the intervention in their in-person treatment sessions. Besides content-related support, therapists also mentioned several practical barriers that the organization should address, such as a slow internet connection.

**Table 10:** Subcodes of the main code “organization,” their definitions, illustrative quote, number of interviews quote was mentioned (N_int_), and number of times code was mentioned (N_tot_).

Subcode	Definition	Illustrative quote	N_int_	N_tot_
Introduction of technology to therapists	Activities that the organization undertook to introduce the technology and train therapists’ necessary skills and knowledge, according to therapists	*Hmm, much less, because, I think I had a course once, just in the beginning, and there’s not really a follow-up. (pp. 10)* *We’ve received a very clear explanation, I believe an entire day and you just started practicing. And I liked that. But now I’m thinking, if I’d have to use it with a patient, I’d really have to ask with the people who gave the course. He’d have to quickly explain to me how it works. That knowledge has faded. (pp. 1)*	17	27
Providing support for therapists	Ways in which the organization offers content-related support to therapists for using the technology in treatment	*I just think that, well, for the long term the organization has to pay more attention to it, for the team. And I also think that you have to implement the trainer-trainer idea. That you pick a couple of people who work well with it or are a bit better in it, that they are appointed as a source of information, and that other people know about that. (pp. 9)* *Currently I’m trying to figure it out myself, but how do you really shape a blended treatment? If there would be education about that! Also about the more challenging cases, if there is very little motivation. (pp. 4)*	14	34
Integration in organizational structures	Extent to which a technology is structurally featured in activities or products for which the organization is responsible, such as meetings, treatment protocols, targets, or performance reviews	*But it doesn’t get indicated a lot in the intake or in meetings. Often the one who’s done the intake has to bring it up themselves, but it’s not something that other people in the meeting bring up or come up with, that that’s also an option. My personal experience. If I bring it up, they say “Oh yeah it’s a good idea,” but if I say nothing, they hardly ever come up with it. (pp. 18)* *So that management says: “eMental health guys, don’t forget about that!” (pp. 6)*	11	21
Providing necessary conditions for use	Extent to which the organization ensures boundary conditions such as availability of sufficient technological resources and time for therapists	*Internet is often slow. Then you’ve planned half an hour, and you think, I’m going to do it. Well, it doesn’t work. It’s also a technical issue. From the division around it, the internet, that’s so slow. And if you have to start a program such as [the intervention], well, you can do something else in that time... Then you constantly have to wait, well, then I drop out. (pp. 2)* *And that time is actually scheduled for people. You have to really work on it, so it cannot disappear in the other activities, because you will forget it. If you really have an hour to only work on eMental health, it will remove some barriers and other arguments. (pp. 18)*	7	10

##### Wider System

This main code refers to the influence of activities by the broader context on use of technology by organizations and therapists and consists of 2 subcodes, which can be found in Table 11. This code was identified 17 times in 11 interviews. Therapists discussed the wider system less often than previous codes. Several participants briefly mentioned health insurance companies and government but did not elaborate on the role of the wider system.

**Table 11:** Subcodes of the main code “wider system,” their definitions, illustrative quote, number of interviews quote was mentioned (N_int_), and number of times code was mentioned (N_tot_).

Subcode	Definition	Illustrative quote	N_int_	N_tot_
Demands of health insurance companies	Therapist perception about financial incentives for using the technology offered by health insurance companies	*I don’t know, that’s something from the health insurer, that they say that we have to do something with [the intervention]. That’s a bit how it feels. That we received that assignment because we have to meet the numbers. (pp. 7)*	9	12
Encouragement of government	Extent to which use of a technology is encouraged by the government	*First, they should make a statement as an organization, together with other organizations, to say: “Yeah it’s all good and you can want it in this way but we’re going higher to the government and say to the government: this is not ok.” There are so many administrative tasks that don’t... If you do something and you have to account for this it’s fine, but it can be a lot easier. (pp. 12)*	4	4

##### Condition

This code refers to the extent to which the nature of the patient’s condition (or psychiatric disorder) influences use of the technology according to therapists. The subcode was mentioned 4 times in 3 interviews, as can be seen in Table 12. Therapists often did not discuss their patients’ diagnoses as a separate factor that directly influences the intervention’s use.

**Table 12:** Subcodes of the main code “condition,” their definitions, illustrative quote, number of interviews quote was mentioned (N_int_), and number of times code was mentioned (N_tot_).

Subcode	Definition	Illustrative quote	N_int_	N_tot_
ADHD^a^	Impact of (symptoms of) ADHD on patient’s use of the technology	*So I cannot really... I do know that those ADHD patients, that it takes too long for them. They’re too easily distracted, or find a question too difficult. It’s not that everyone has that. (pp 2)*	3	4

^a^ADHD: attention deficit hyperactivity disorder.

### Synthesis

When looking at the implementation strategies described by Waltz et al [19], desk research and interviews showed that in this study, most attention was paid to training of therapists, as mostly becomes clear in the subcode *introduction of the technology* *to therapists* of the main code “organization.” However, little to no strategies related to changes in the organizations’ infrastructure, engagement of patients, and adaptation of technology to the context were conducted. This is further illustrated by all subcodes of the main code “organization,” but also by the therapist- and patient-related subcodes *integration of technology in routines*, *knowledge and skills*, *discussing technology with colleagues*, and *motivation*. Furthermore, desk research showed that support and assistance for therapists was available, but most interviewed therapists did not experience this as such, which becomes most clear in the “organization”-related subcode *providing support for therapists*. Desk research showed that besides training, several relatively minor evaluation strategies were conducted by the organization itself, during and shortly after the pilot. However, the outcomes of these evaluations did not lead to major changes to the implementation strategies, so no lasting improvements in the use of the intervention were observed in the log data. This is visualized in Figure 4; a short peak in sent messages can be observed during the time of the evaluation, but this increase in sent messages only lasted several months. To conclude, implementation strategies were mostly focused on training of therapists, but little attention was paid to adaptiveness of the technology, changes in the organization, and patient awareness.

The results of the desk research, log data analysis, and interviews were used to assess the implementation outcomes described by Proctor et al [21] from a holistic perspective, structured via the NASSS framework [31]. First, the interviews showed that acceptability of the intervention was relatively high, with therapists being positive about the intervention and able to identify its added value. This fairly high acceptability is illustrated in Table 4, which shows overall good first impressions of the intervention, and is further supported by the main code “values,” which points out that therapists are able to mention a broad range of potential and actual advantages. However, despite the fairly positive acceptability, log data analyses and interviews clearly showed that adoption was low; a large share of therapists and patients did not use the intervention at all. Furthermore, the intervention’s penetration in the organization was low; log data showed that only a small fraction of therapists and patients used the intervention. Only 54% of the eligible therapists actually used the intervention, and Figure 4 shows that the largest share those who did use it, did not use it a lot. When the intervention was used, fidelity was often low, as can be seen in Table 2. Only 18% of the modules were fully completed, and of the remaining 82%, modules were either not started or not fully completed, implying that they were not used as intended. An explanation for this can be found in the appropriateness of the intervention. Log data showed that several modules were completed, and several therapists indicated that they were able to successfully use the module with some patients. However, therapists indicated that the intervention did not optimally fit most patients’ skills and preferences. This is illustrated by the patient-related subcodes *conscientiousness*, *literacy and education level*, *technological skills*, and *reflective skills*. The mismatch between patient characteristics and the intervention also becomes clear in the main code “technology,” which shows that usability, design, and content are not optimally tailored to the forensic psychiatric patient population. Furthermore, the intervention also seems not to be appropriate for many therapists, who indicated that they prefer in-person contact and often felt not fully equipped to integrate the intervention in their treatment. This means that currently, the intervention’s costs in terms of finances and time investment seem to be higher than the benefits. Also, sustainability was low: therapists stated that the intervention was often not discussed in meetings and was not integrated in electronic patient records they used. While this theme appears in multiple codes, it becomes especially clear in the organizational subcode *integration in organizational structures*. Consequently, therapists often did not even think of the possibility to use the intervention in treatment, which is represented by the therapist-related subcode *integration of technology in routines*. Currently, the feasibility of the intervention is low because of a suboptimal fit between the features of the technology; needs, wishes, and skills of therapists and patients; and characteristics and activities of the organization. It appears that since the implementation strategies were not conducted from a holistic perspective but mainly focused on training the therapists, the implementation outcomes are disappointing.

## Discussion

### Principal Findings

#### Main Outcomes

This mixed-method study evaluated the implementation strategies and outcomes of an eMental health intervention in forensic mental health care from a holistic perspective, where attention is paid to factors related to people, organizational context, and technology. Triangulation of the outcomes of desk research, log data analyses, and interviews with therapists showed that the technology did not optimally fit the therapists, patients, and organization. Furthermore, the implementation process was mostly focused on skill training of therapists and not executed from a holistic perspective; not enough attention was paid to changes in the organization, patients, and other required changes in therapists. The results of this mixed-methods study will be discussed in more detail structured by the main elements of the holistic approach that was applied: the people using the eMental health intervention, the organization in which it was used, and the technology.

#### Therapists

The interviews and log data showed that although several therapists were active users of the intervention, most of them only tried it once or twice, and a relatively large share of the therapists did not even use the intervention at all. Nevertheless, almost all interviewed therapists were fairly positive about the intervention and able to identify its added value. This shows that cognitions, intentions, and feelings of users are not fully predictive of successful use. Nevertheless, models that focus on individual factors predicting technology acceptance, such as the technology acceptance model (TAM) [46] or the unified theory of acceptance and use of technology [47], are still used regularly to analyze or plan implementation. While these types of models are useful to create an overview of individual beliefs and attitudes that influence a person’s intention to use a technology [48], they pay little to no attention to influences of the context, characteristics of the technology, and interrelationships between them [48,49]. Consequently, implementation models or frameworks that apply such a holistic approach like CFIR [30] or the NASSS framework [31] seem to be more useful in this context because of their focus on a broad range of contextual and (inter)personal factors and not merely individual factors of end users.

Despite the fact that all therapists in the organization had received training, only a relatively small proportion actively used the intervention. This implies that skills training only did not suffice for successful implementation: more than just a how-to instruction seems to be necessary to fully equip therapists to embed the intervention in their treatment sessions. Among other things, therapists also need to know how to persuade patients to start with the intervention, they need to be able to keep motivating patients to complete exercises, and they must embed the content of the intervention and the patient’s answers in treatment [14,50,51]. This implicates that the use of eMental health might also change the role of the (forensic) mental health professional [51]. In this new way of working, patients might be more in the lead and supported by professionals, and the structure and content of treatment may not be determined only by the professional but also by the intervention. Such a technology-induced role change in domains where communication previously only took place between persons requires changes on a multitude of levels, like management, education, or government [52], and not merely a skills training of therapists. More research on the nature of this role change and implications for implementation strategies is required.

When looking at implementation strategies, it might also be useful to conduct more research on the need to better tailor these strategies to different types of therapists. In this study, there appeared to be a lot of differences in therapists regarding their subjective attitudes and objective use of the intervention. This is in line with a recent study that showed that therapists differ in the types of drivers and barriers they perceive with regard to the use of eMental health [24]. This might imply that different types of therapists benefit from different types of implementation strategies. For example, therapists with a low level of enthusiasm and skills might need to receive a different type of training than enthusiastic and tech-savvy therapists. In line with this, multiple researchers stated that one-size-fits-all interventions are not very suitable for forensic mental health care and that tailoring is advised [40,53-57]. This argument can be extended to professionals: adaptive implementation strategies that fit different types or subgroups of therapists’ needs, skills, and attitudes might be beneficial for implementation outcomes. Subsequent research might focus on the identification of different subgroups of therapists, for example, in terms of attitude or eHealth literacy and tailoring implementation strategies to these characteristics. Also, researchers should assess whether tailoring implementation strategies to different types of professionals actually results in better implementation outcomes.

#### Patients

Therapists indicated that the eMental health intervention requires a relatively high level of reading and writing skills, cognitive reflection, and conscientiousness. However, according to desk research and other literature, forensic psychiatric patients often have low education levels [58,59], which might explain the low number of patients that completed modules. This shows that there seems to be a poor fit between most users’ skills and the content of the eHealth technology, which might be a major cause for nonuse or nonadherence [60,61]. Several solutions might address this issue: therapists can support patients more in working on difficult elements of the intervention, or texts and assignments can be shortened and made easier. However, it might also be possible that the studied eMental health intervention is not very suitable for this context and another type of technology would be a better fit for most patients. For example, multiple recent studies point out the potential of interactive virtual reality interventions for forensic mental health care [62-66], among other things because they allows patients to actually practice with behavior instead of talking or writing about it. Another possibility is wearables, which can be used to collect physiological data associated with aggressive outbursts or as electronic momentary assessment devices to gather information about a patient’s emotional state [67,68]. This study underlines the importance of adaptability of eMental health to optimally fit the needs and characteristics of individual patients.

#### Organization

As was mentioned before, the organization focused implementation strategies mostly on skills training of therapists but did not pay much attention to the implementation of the intervention on other levels. The disappointing implementation outcomes show the importance of the use of multiple types of implementation strategies to ensure that an eMental Health intervention is thoroughly embedded in a forensic organization’s infrastructure [40,50]. This is in line with literature on eHealth implementation in general, which emphasizes the importance of integrating technologies in existing organizational structures or even changing the way care is delivered or organized [28,29,32,33,69]. One way to achieve this in the studied organization is by ensuring that therapists structurally discuss the possibility of using an eMental health intervention at predetermined moments in treatment (eg, during a patient’s intake). This can be done by means of the existing “fit for blended care” instrument [12], which aims to support therapists in shaping their blended treatment in cooperation with the patient. This instrument can be adapted to fit the specific forensic mental health organization by means of the patient-related factors identified in this study. Furthermore, the eMental health intervention might need to become a permanent item on the agenda of team meetings to ensure that it is discussed regularly [50]. Moreover, therapists indicated that they hardly discussed the intervention with colleagues, as opposed to other parts of their treatment, so peer-coaching sessions to discuss the use of the intervention might be organized [50]. It is important that research is conducted to determine whether these types of strategies actually boost implementation outcomes [23]. Generating more knowledge on suitable and successful implementation strategies will support other organizations in planning implementation and prevent them from reinventing the wheel, which will eventually save time and money.

#### Technology

One reason for the low use of the intervention was that not all therapists were positive about the user-friendliness of the intervention’s design: among other things, they indicated that the website did not give them a clear overview of suitable modules for specific patients. Log data indeed showed that only a fraction of modules were used frequently. Adding more persuasive elements to the intervention might support therapists in using the intervention. An example is tunneling: the system can guide the therapist through the process of selecting suitable modules for a patient [70]. Additional research can be conducted to evaluate and improve the persuasiveness of the intervention (eg, by means of the Perceived Persuasiveness Questionnaire [71]), which might increase its use [72].

A characteristic of the intervention that might have hindered use is the lack of possibilities for personalization. As was mentioned before, the technology does not seem to fit most patients according to therapists: there were too many sessions within a module, there was too much text, and the subject matter was too complex for most patients. Therapists expressed the need for multiple versions of the modules in order to personalize the intervention. Examples are the possibility to choose between videos or text or the option to select texts with different levels of difficulty. Studies on eHealth in general have stated that personalization can increase adherence [73-75], so a more personalized version of the intervention might result in better implementation outcomes. However, more research is necessary on what elements should be personalized, how this should be done, and if this actually positively impacts use and adherence. Ideally, this redesign of the technology should be done in close cooperation with end users to ensure that it better fits their needs [29,64], since cocreation can also have a positive influence on implementation outcomes [28,76].

### Strengths and Limitations

This study took place at one forensic psychiatric hospital in the Netherlands, which might raise questions about the generalizability of the results. However, other studies on eHealth in forensic care have identified similar types of implementation issues [50,53,56,63,77]. On top of that, many of the identified issues have been reported for eHealth in general (eg, lack of enthusiasm in therapists, low adherence by patients, or lack of integration of a technology in an organization’s structure [32,33,69]). This implies that, on an abstract level, our findings are relevant for other types of (mental) health care as well. A related strength of this study is that all therapists working at the outpatient clinic could be interviewed, which prevented a self-selection bias from occurring.

Furthermore, while use of the NASSS framework to structure the qualitative analysis was a strength of this study, several issues arose during the coding process. A chief example of this is the domain “condition.” As opposed to a patient population that suffers from one disorder (eg, depression or diabetes), the forensic psychiatric population is not characterized by one condition: comorbidity is very common among forensic patients, and patients have committed a broad range of offenses [78-80]. This raises the question on how to characterize certain behaviors or cognitions: as part of a patient’s personality or as a symptom of a disorder. To illustrate: if a patient has trouble focusing, which hinders the use of an eHealth intervention, should this be viewed as a consequence of the patient’s ADHD or as a part of their personality? We therefore recommend that more studies apply the NASSS framework to evaluation of implementation and report on its applicability and suitability, which might lead to possible revisions or fine-tuning of the framework.

Finally, a strength of this study was the combination of different types of data to evaluate the implementation process. By using a mixed-methods approach, objective and more subjective data were combined, which proved to be valuable for gaining insight into a multilevel and elaborate implementation process. It was decided to not interview patients and management, since therapists were asked about the patient perspective and desk research provided insight into the strategies of management. Including these perspectives in interviews would probably not have produced much new information, but it is possible that factors were overlooked. Further research could focus on analyzing the patient perspective in implementation and investigate whether there are any discrepancies between therapist perspectives on patients and the patients’ own perceptions. Finally, desk research was combined with interviews to provide a full picture of the implementation strategies, but not all information could be retrieved from the desk research (eg, number of participating therapists in the pilot). This shows the importance of carefully and fully documenting relevant information about implementation strategies from the start.

### Conclusion

This study showed that the fit between the characteristics and needs of the therapists and patients, the organization, and the technology was suboptimal, which has led to suboptimal implementation outcomes. An explanation for this could be the lack of a holistic approach in implementation: the implementation strategies mainly focused on training therapists’ technical skills, while more attention should have been paid to necessary changes in the organization, an attitude change in therapists, and design of the technology. Here, adaptivity appears to be an important concept: a technology should be easily adaptable to an individual patient, therapists should be trained to be able to deal with an eMental health intervention in their treatment in a flexible way, and organizations must adapt their implementation strategies and structures to embed a new eHealth intervention. Consequently, in implementation, the holistic nature of eHealth and ensuring adaptivity on multiple levels appear to be pivotal.

## Abbreviations

ADHD: attention deficit hyperactivity disorder
CFIR: consolidated framework for implementation research
eMental health: technology in mental health
NASSS: nonadoption, abandonment, scale-up, spread, and sustainability
